# Adolescents in a residential school for behavior disorders have an elevated mortality risk in young adulthood

**DOI:** 10.1186/s13034-015-0078-z

**Published:** 2015-09-02

**Authors:** Marko Manninen, Maiju Pankakoski, Mika Gissler, Jaana Suvisaari

**Affiliations:** National Institute for Health and Welfare, Mannerheimintie 166, 00300 Helsinki, Finland

**Keywords:** Juvenile delinquency, Mortality, Child welfare

## Abstract

**Background:**

Conduct problems during adolescence are associated with an elevated mortality risk. This study investigated the mortality rate, causes of death, and changes over time in a Finnish residential school (RS) population.

**Methods:**

All adolescents (N = 885, M/F = 594/291, age mean 15.2 years at baseline) residing in the RS system in 1991, 1996, 2001, and 2006 and matched controls were included in a register-based study with a follow-up time of up to 22 years.

**Results:**

The all-cause mortality rate for people with an RS background was 6.7 % compared to 1.0 % in the controls (Hazard Ratio HR = 6.95, 95 % 4.66–10.37, p < 0.001). 8.1 % of the RS boys had died compared to 2.2 % of the girls (HR = 2.2, p = 0.02). The HR for substance-related death was 24.31 (95 % CI 9.3–65.53, P < 0.001), for suicide 7.23 (95 % CI 3.24–16.11, P < 0.001) and for other external causes 5.45 (95 % CI 2.41–12.36, P < 0.001) compared to controls. Mortality peaked among RS boys at approximately 25 years, whereas for girls it peaked after 30 years.

**Conclusions:**

Adolescents with severe disruptive behavior problems have a seven-fold risk for premature adult-age death compared to matched controls. The most common causes for death were avoidable, substance-related followed by suicide. Effective treatment of mental and substance use related problems during and after the placement is needed to reduce mortality.

## Background

Residential schools (RS) in Nordic countries are child welfare institutions for adolescents with severe conduct problems. The adolescents placed in RS have disruptive behavior spectrum problems, which typically include juvenile delinquency, substance use, and severe school dysfunction [1]. In Finland in 2011, there were 14 783 children and adolescents (1.4 % of the population aged less than 18 years) placed outside the home by child welfare services, and 274 (1.8 %) of these resided in eight residential schools [2]. The median age for RS placement is 15 years, and the placement ends at the age of consent (18 years), after which the adolescents are provided a voluntary 5-year after-care program [1].

The RS system is a part of child welfare, not the correctional system: the focus in the RS placement is therefore rehabilitation, not punishment. For example, education has a high priority, and all residential school adolescents in recent years have completed compulsory education. Over the past two decades, RS have been systematically developed to meet the needs of RS adolescents, who often have mental health and substance use problems [3–5] as well as cognitive difficulties [6]. Despite the intensive intervention provided to the adolescents, previous small-scale short-term follow-up studies have shown that psychiatric treatments and criminal behavior are common after the placement [7, 8].

The association between childhood and adolescent conduct problems with an increased mortality risk has been observed in numerous population-based cohort studies [9–11]. Studies with long follow-up time—up to 50 years [12] or 60 years [13]—have confirmed the association. The standardized mortality rate (SMR) associated with oppositional defiant disorder, conduct disorder, or substance use disorder diagnoses is four-fold compared to the population [14], while the SMR among people using mental health services is two-fold [15–17]. Furthermore, young offenders sentenced to custody have an SMR of 9.4 for men (95 % CI 7.4–11.9) and 41.3 for women (95 % CI 20.2–84.7) [18]. In Finland, a study of young male offenders sentenced to prison showed an SMR of 7.4 (95 % CI 6.7–8.1) [19]. This correlation between disruptive behavior and excess premature mortality appears to remain throughout life: a meta-analysis on adult criminal studies show SMR ranges from 1.0 to 9.4 for males and 2.6 to 41.3 for females following release from prison [20].

The causes of death associated with disruptive behavior differ from those of general population. In its most extreme form this is seen in young offenders, among whom the most common causes of death are drug-related (SMR 25.7), suicide (SMR 9.2) and non-intentional injuries (SMR 5.7) [18]. The association between conduct problems and substance abuse is also a well-replicated finding [10, 21–23]. In Finland, substance use disorders are associated with a 3- to 50-fold risk of death, and these deaths are most commonly due to opioid use [24]. The risk for substance-related death is even higher when conduct disorder is accompanied with depression [25], and both substance use problems and mood disorders are common among RS adolescents [3, 4].

Conduct problems are also associated with increased risk of suicide [22]. For example, a large-scale Finnish population follow-up study by Sourander et al. [11] linked conduct and conduct-emotional problems at the age of 8 with an elevated risk for suicide in adolescence and young adulthood (OR 6.2, CI 1.8–20.9). Death by non-intentional injury is likewise over-represented in the delinquent population; the excess number of accidents has been proposed to reflect poor self-care, and accident-proneness is also intertwined with substance use [12].

The risk for premature death appears to decrease with time [19]. Despite this proportional decrease, the trend of increased mortality among the delinquent subjects continues at least until age of 65 [12]. Moreover, the results from the same follow-up study by Laub et al. also showed that age has an effect on the causes of death: 76 % of delinquents’ premature deaths before the age of 40 were due to external causes of injury or poisoning—namely accidents, suicide, homicide, and substance-related events—while after the age of 40, these causes were accompanied with excess deaths due to diseases and medical causes.

In Finland, the number of children placed outside the home has doubled since 1991 [26], and the percentage of adolescents from an immigrant background is growing both in the general population and especially among those entering foster care and RS [1]. These changes pose a challenge to the contemporary clinical procedures in use in the RS, but the lack of reliable, large-scale follow-up studies make developing the current system difficult.

Taken together, the current literature suggests that adolescents referred to RS due to severe behavioral problems may have an elevated risk for premature death, but this has not been investigated previously. Moreover, it is not known whether the recent changes in the socio-economical and cultural background of the Finnish adolescent population, or the systematic efforts to develop education, treatment, and rehabilitation provided in the reform schools are reflected in the long-term outcome of adolescents placed in RS. This study compares the mortality of adolescents placed in RS to a matched general population control group in a register follow-up of up to 22 years. The specific aims of the study are to compare mortality risk by main causes of death in residential school adolescents and controls, to assess whether the mortality risk and causes of death in RS adolescents have changed over time, and to assess whether there are excess mortality peaks shortly after the placement has ended or later.

## Methods

The RS adolescents (N = 885, M/F = 594/291, age mean 15.2 years at baseline) were identified from the Finnish welfare registry kept by the National Institute for Health and Welfare (Terveyden ja hyvinvoinnin laitos, THL). The inclusion criterion was out-of-home placement status *residential school* on the last day of the year in 1991, 1996, 2001, or 2006: the data acquired were thus organized into four cohorts. These four cohorts were selected for the investigation of changes in the outcome of RS adolescents over time. As 5-year intervals were used, the majority of children were only in one cohort in the original data. The children with entries in more than one cohort were removed from the later one. The birth years ranged from 1973 to 1994. The controls (N = 4316) were chosen by the criterion of having no RS placement entries and matched by sex, age, and place of birth (municipality) with the RS adolescents. The aim was to get five matched controls for each case, which was not possible for 71 residential school adolescents (6 % of all cases). This was due to difficulties in finding suitable controls, for example if the RS adolescent had been born in a small municipality. In the final data, 58 cases had four matched controls each, and the remaining 13 one to three controls each. All RS children were included in the study, regardless of the final number of controls. The study protocol was reviewed and approved by the institutional review board of the National Institute for Health and Welfare, Finland.

Mortality data was obtained from the Causes of Death Register, kept by Statistics Finland. The data are based on death certificates, and the coding of causes of death is controlled by medical experts at the local level and at Statistics Finland [27]. The causes of death were categorized in five groups: Substance-related deaths, Suicide, External causes, Diseases/medical conditions, and Unknown. *Substance*-*related deaths* refer to alcohol- and drug-related deaths, as defined by Nordic Medico-Statistical Committee (NOMESCO) guidelines [28]. The category label *Unknown* refers to deaths which have occurred abroad, so that the Finnish authorities were not able to determine a cause of death. For diagnoses in 1991–1995, the corresponding ICD-9 codes were used for categorization. The mortality data acquisition date was 11th of November 2013, and the follow-up time after residential school ranged from 1 to 22 years.

Survival analysis was conducted with stratified Cox regression, which accounts for the matching of individuals within the groups of one RS adolescent plus matched controls. The four residential school cohorts were compared to each other by Kaplan–Meier survival analysis. The Hazard Ratios (HR) comparing residential school subjects and controls with respect to different causes of death were calculated by stratified Cox regression. The percentages for different causes of death among the RS population were also reported cohort-by-cohort. Mortality hazard rates were calculated for different age categories. Smoothed curves were obtained by a kernel-like smoothing procedure, and mortality hazard rates were calculated in R version 3.1.1 package muhaz version 1.2.6. [29]. Survival analyses were performed using IBM SPSS Statistics version 21.

## Results

### Mortality

The risk for premature death in RS adolescents was seven-fold (HR = 6.95, 95 % CI 4.66–10.37, p < 0.001) and was similar for males (HR = 6.93, 95 % CI 4.46–10.75, p < 0.001) and females (HR = 7.05, 95 % CI 2.68–18.53, p < 0.001). The difference was largest in the 1991 cohort: 14.6 % (M/F 16.7 %/8.3 %) of RS adolescents had died during follow-up compared to 1.5 % (M/F 1.4 %/1.7 %) of controls. The mortality rate for RS adolescents and their controls by cohort and sex is shown in Table 1.

**Table 1:** Deaths for residential school (RS) population and controls by cohort and sex. As the follow-up times vary, the numbers are not comparable between the cohorts

Cohort		Males		Females		Both	
		RS	Controls	RS	Controls	RS	Controls
1991	N (deaths/all)	25/150	10/722	4/48	4/239	29/198	14/961
		16.7 %	1.4 %	8.3 %	1.7 %	14.6 %	1.5 %
1996	N (deaths/all)	11/142	11/689	5/64	2/313	16/206	13/1002
		7.7 %	1.6 %	7.8 %	0.6 %	7.8 %	1.3 %
2001	N (deaths/all)	10/143	8/697	0/74	1/363	10/217	9/1060
		7.0 %	1.1 %	0.0 %	0.3 %	4.6 %	0.8 %
2006	N (deaths/all)	3/159	6/783	1/105	0/510	4/264	6/1293
		1.9 %	0.8 %	1.0 %	0.0 %	1.5 %	0.5 %
Total	N (deaths/all)	49/594	35/2891	10/291	7/1425	59/885	42/4316
		8.2 %	1.2 %	3.4 %	0.5 %	6.7 %	1.0 %
	HR (95 % CI)	6.93 (4.46–10.75)		7.05 (2.68–18.53)		6.95 (4.66–10.37)	

**Fig. 1:**
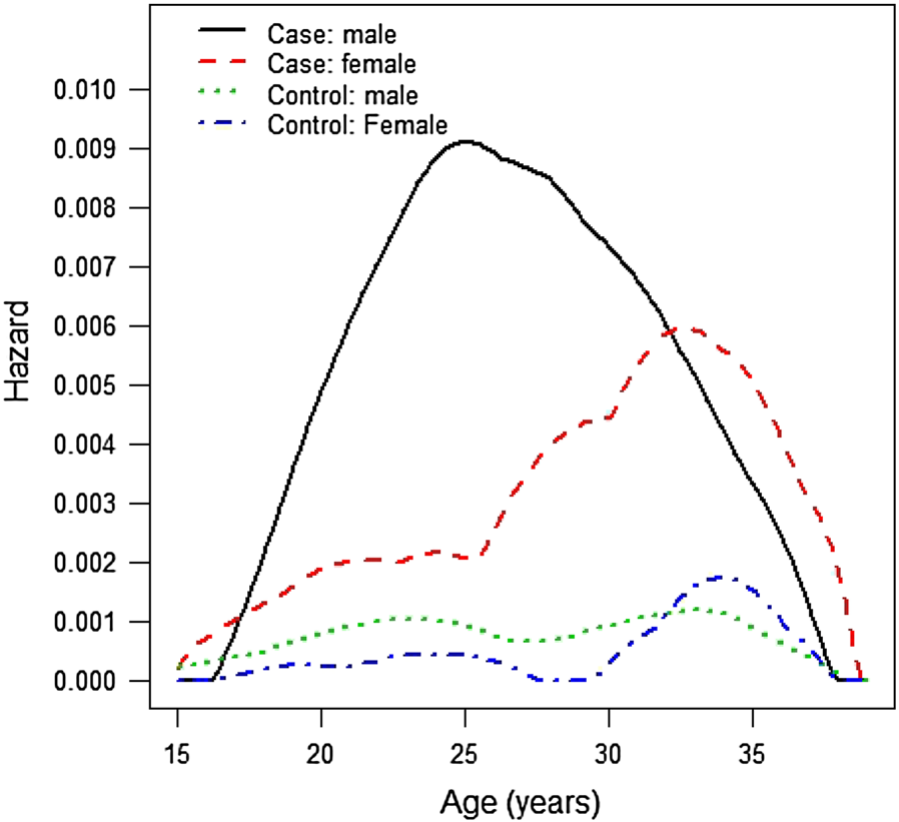
Mortality hazard functions for residential school population and controls by sex (N = 5201). Globally optimal estimates

**Fig. 2:**
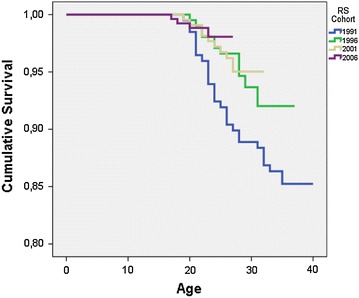
Kaplan–Meier survival curves for the four RS cohorts

Mortality hazard functions for RS subjects and controls by age and sex are presented in Fig. 1. The mortality hazard function for RS boys peaked at approximately 25 years of age, whereas for RS females it peaked after 30 years. The Kaplan–Meier survival plot comparing RS cohorts suggested a difference between the 1991 cohort and the later ones (Fig. 2), but the difference did not reach statistical significance (p = 0.168).

### Causes of death

Table 2 presents the categorized causes of death for RS subjects and controls, as well as the HRs for each category. The elevated risk for substance-related death was 24-fold, for suicide seven-fold, for death by external causes five-fold, and for death by unknown cause eight-fold. All these differences were statistically significant. Of the 12 deaths due to external causes, eight (67 %) were traffic accidents. Mortality related to diseases and medical conditions was not elevated in RS population (HR 0.49, 95 % CI 0.01–3.82).

**Table 2:** Causes of death for residential school population and controls

Cause of death	RS		Control			95 % CI		
	N	%	N	%	HR	Lower	Upper	Sig.
Substance-related	25	42.4	5	11.9	24.31	9.30	63.53	***
Suicide	16	27.1	10	23.8	7.23	3.24	16.11	***
External causes	12	20.3	12	28.6	5.45	2.41	12.36	***
Disease or medical condition	1	1.7	12	28.6	0.49	0.06	3.82	NS
Unknown	5	8.5	3	7.1	8.33	1.99	34.87	**
Total	59	100	42	100				

*HR* hazard ratio, *NS* non-significant

*** p < 0.001; ** p < 0.01

The sex differences in causes of death are shown in Table 3. Nearly half of the deaths among RS males were substance-related (alcohol and drugs together 46.9 %), followed by suicide (26.5 %), external (20.4 %), unknown (4.1 %), and medical (2.0 %) causes. One RS male died from homicide. For RS females, the most common cause of death was suicide (30 %), followed by substance-related (20 %) and external causes (20 %). In addition, 30 % of RS females’ deaths were due to unknown causes. Due to the small group sizes, statistical testing by gender was not done.

**Table 3:** The categorized causes of death for residential school (RS) population and controls

Sex	RS (N = 885)			Controls (N = 4316)		
	N	% of RS population	% of deaths	N	% of control population	% of deaths
Male						
Substance-related	23	3.8	46.9	5	0.2	14.3
Suicide	13	2.2	26.5	9	0.3	25.7
External cause	10	1.7	20.4	10	0.3	28.6
Disease	1	0.2	2.0	8	0.3	22.9
Unknown	2	0.3	4.1	3	0.1	8.6
Total	49	8.2	100	35	1.2	100
Female						
Substance-related	2	0.7	20.0	–	–	–
Suicide	3	1.0	30.0	1	0.1	14.3
External cause	2	0.7	20.0	2	0.1	28.6
Disease	–	–	–	4	0.3	57.1
Unknown	3	1.0	30.0	–	–	–
Total	10	3.4	100	7	0.5	100

## Discussion

Adolescents placed in residential schools have a substantially elevated mortality risk in young adulthood: the results from this study show a seven-fold overall risk for death. All excess mortality was due to substance-related causes, suicide, or external causes, whereas mortality from diseases/medical conditions was not elevated. These figures are higher than those found among patients with mental disorders (SMR 2.22, 95 % CI 2.12–2.33) [17], and they are also higher than those from population studies addressing disruptive behavior disorder (SMR 5.0) [30]. The mortality rates found in this study resemble the figures found among young offenders sentenced to prison [18, 19], and those found in adult prison studies [20, 31].

The mortality hazard is age-dependent. The difference in mortality between RS population and controls begins to widen as the age of consent (in Finland, 18 years) is reached and adolescents leave the RS system, but premature mortality peaks later, at about 23–28 years of age among men and after age 30 among women. This is different from, for example, results from young offenders, among whom the mortality risk appears to peak during the first weeks after release from prison [31]. In the current RS service system, the provided after-care programs stop 5 years after the age of consent, which translates to 23 years of age. As the mortality risk in RS males peaks at 23–25 years, it appears that the cessation of after-care is a critical period for young males from an RS background. For RS females, the relative mortality risk peaks even later, after 30 years of age. Their elevated risk might be connected to problems related to family relations or child bearing, but the current data are insufficient to analyze this in greater detail. Further research is needed to disentangle the gender-specific risk factors. Nevertheless, our results suggest that there is a need for long-term support after the official after-care program ends.

The death rate in the 1991 RS cohort was higher than in other cohorts, but the difference did not reach statistical significance. A plausible explanation for this trend is the financial depression in Finland in the early 1990’s, which led to cuts to funding for the welfare system. Even though there were differences between municipalities, it is possible that the after-care for the 1991 cohort was considerably less extensive than for the younger cohorts.

Substance use was the single most common cause of death within the RS population. The risk for premature death due to substance use was 24-fold compared to controls. These substance-caused mortality rates are higher than those associated with opioid use (SMR 14.7) [30], or with alcohol use disorder (mortality rate ratio 3.0–5.2) [32] in the general population. The figures found in this study again resemble the findings from studies on adult criminals, in which substance use is the leading cause of death (SMRs 4.1–26) for released adult prisoners, accounting for 18 % of premature deaths [20]. The association between a history of governmental care and substance use disorders (SUD) has been reported from other countries as well [33]. Our results suggest that interventions for preventing SUDs should be an integral part of RS treatment.

The suicide mortality rate in the RS population was seven-fold compared to the controls, which corresponds to the rates found in prisoners [20] and approaches that found in severe mental disorders and hospital-treated substance use disorders [29, 32]. Psychiatric disorders are common in RS adolescents [3, 4], and should be identified and treated when the adolescents are in residential care. Furthermore, school-based suicide prevention programs [34] should be implemented in reform schools. Nevertheless, the fact that mortality risk peaks several years after the RS placement has ended suggests that RS adolescents need continuing support and care in young adult life. A history of severe disruptive behavior problems should be recognized as a risk factor for suicidal behavior.

External causes were the third most common causes of death. The majority (67 %) of these deaths were caused by traffic accidents. Further, it is difficult to assess how many of the traffic deaths were suicides. The current literature suggests that 2–6 % of traffic deaths are intentional [35, 36]. Comorbid substance use disorder and/or intoxication were found in two thirds of the traffic-related deaths, which again emphasizes the key role of substance-related problems.

Adult prisoners are known to have an excess of both physical problems and psychiatric disorders [30, 37]. In this study, however, there was no excess risk due to diseases and medical conditions. This was probably due to the relatively young age of the subjects: physical health problems typically accumulate at older age.

In the United States, adolescent delinquents have a high risk for homicide victimization [38], whereas there was only one homicide victim among the 59 RS deaths in this study. In Finland, youth gang membership and gun violence are rare, and typical homicides take place among middle-aged, unemployed, alcohol-dependent men [39].

It has been said that prison provides a public health opportunity to treat both physical and psychiatric problems which might not be treated in the community [37]. Likewise, residential school has been described as a second chance, as placement facilitates effective interventions before adulthood [3]. The results from this study suggest that especially RS males would benefit from an intensive, long-term after-care lasting until the early thirties. However, working with delinquent adolescents might prove difficult: in addition to their multiple and intertwined problems, these adolescents might have a hostile attitude towards the personnel and the whole care system. This is especially true in the after-care, in which the drop-out rates appear to be high. According to the RS personnel, the main reason for poor commitment is the lack of personal, long-term relationships between the adolescents and after care personnel. A trusting relationship is, unfortunately, difficult to achieve due to a high turnover of after-care workers.

The strengths of this study include a sufficiently large RS population and an extensive follow-up data from Finnish registries without drop-outs. The large data set makes the findings reliable. The most obvious limitation concerns generalizing the results. The Finnish residential school system differs from similar institutions in other countries: it is a part of the welfare, not the juridical system, and the placement decision is influenced by unique factors. For example, in our data set it remains unclear which adolescents fulfilled the diagnostic criteria for conduct disorder or substance use disorder during placement. However, RS placement *per se* is an indication of severe behavioral problems. Another limitation is the lack of data on the socioeconomic status (SES) of the subjects and controls: low SES is a well-known factor affecting life expectancy in the general population [40, 41], but obtaining reliable SES information for foster-care RS adolescents was not possible. Moreover, female deaths were rare, which results in weak statistical power: female-only interpretation of the results should be done with care. Taken together, these limitations do not change the main outcome of this study: the high mortality rate among former RS adolescents calls for immediate action.

Early adulthood is a critical period for emerging health inequalities, and the ultimate outcome measure for health inequalities between population subgroups is premature death. Delinquent adolescents’ problems resemble those of adult prisoners’, but in the main their problems are less severe and less intertwined, and thus the prognosis should be better. Interventions targeting mental health and substance use should be provided during the residential school placement, but our results also suggest that continuing the open care programs after RS is crucial. These adolescents need long-lasting and multi-faceted support in the transition phase from residential school adolescence to being a self-supporting adult member of society. Despite differences between the institutions and welfare policies in Finland and other countries, the results from this study underline substance use and mental health problems as the key factors affecting premature mortality among adolescents with severe conduct problems.

## Conclusions

- Adolescents placed in a residential school for behavior disorders have an elevated risk for premature death in early adulthood.
- Compared to the general population, the difference in mortality begins to widen after the end of placement.
- The premature mortality is mainly due to mental health and substance use problems.
- The excess mortality is a specific public health inequity, which calls for effective screening and intervention procedures.
- Targeted interventions should be provided during placement, and open care programs should continue after RS: these adolescents need intensive support in the transition phase from residential school adolescence to self-supporting adulthood.

## Abbreviations

HR: hazard ratio
RS: residential school
SES: socio-economic status
SUD: substance use disorders
SMR: standardized mortality rate
THL: National Institute for Health and Welfare, Finland (Terveyden ja hyvinvoinnin laitos)
